# Proteome Characterization of BALF Extracellular Vesicles in Idiopathic Pulmonary Fibrosis: Unveiling Undercover Molecular Pathways

**DOI:** 10.3390/ijms22115696

**Published:** 2021-05-27

**Authors:** Enxhi Shaba, Claudia Landi, Alfonso Carleo, Lorenza Vantaggiato, Eugenio Paccagnini, Mariangela Gentile, Laura Bianchi, Pietro Lupetti, Elena Bargagli, Antje Prasse, Luca Bini

**Affiliations:** 1 Department of Biotechnology, Chemistry and Pharmacy, University of Siena, 53100 Siena, Italy; enxhi.shaba@unisi.it; 2 Functional Proteomics Lab, Department of Life Sciences, University of Siena, 53100 Siena, Italy; lorenz.vantaggiato@student.unisi.it (L.V.); bianchi12@unisi.it (L.B.); luca.bini@unisi.it (L.B.); 3 Department of Pulmonology, Medizinische Hochschule Hannover at Fraunhofer-Institut für Toxikologie und Experimentelle Medizin, 30625 Hannover, Germany; carleo.alfonso@mh-hannover.de (A.C.); prasse.antje@mh-hannover.de (A.P.); 4 Department of Life Sciences, University of Siena, 53100 Siena, Italy; eugenio.paccagnini@unisi.it (E.P.); mariangela.gentile@unisi.it (M.G.); pietro.lupetti@unisi.it (P.L.); 5 Respiratory Diseases and Lung Transplantation, Department Internal and Specialist Medicine, University of Siena, Viale Bracci 16, 53100 Siena, Italy

**Keywords:** shotgun proteomics, EVs, IPF, BALF

## Abstract

In the longtime challenge of identifying specific, easily detectable and reliable biomarkers of IPF, BALF proteomics is providing interesting new insights into its pathogenesis. To the best of our knowledge, the present study is the first shotgun proteomic investigation of EVs isolated from BALF of IPF patients. Our main aim was to characterize the proteome of the vesicular component of BALF and to explore its individual impact on the pathogenesis of IPF. To this purpose, ultracentrifugation was chosen as the EVs isolation technique, and their purification was assessed by TEM, 2DE and LC-MS/MS. Our 2DE data and scatter plots showed considerable differences between the proteome of EVs and that of whole BALF and of its fluid component. Analysis of protein content and protein functions evidenced that EV proteins are predominantly involved in cytoskeleton remodeling, adenosine signaling, adrenergic signaling, C-peptide signaling and lipid metabolism. Our findings may suggest a wider system involvement in the disease pathogenesis and support the importance of pre-fractioning of complex samples, such as BALF, in order to let low-abundant proteins-mediated pathways emerge.

## 1. Introduction

Bronchoalveolar lavage (BAL) is a relatively non-invasive procedure used in pulmonary medicine, consisting of washing selected lobes of the lung with saline buffer solution by fiberoptic bronchoscopy and in the recovery of the fluid. This fluid consists mainly of cells, both resident alveolar cells and recruited inflammatory cells, their secreted products and proteins leaked across the endothelial–epithelial barrier. Its cell-free component, commonly referred to as “Bronchoalveolar Lavage Fluid (BALF),” is quite similar in composition to other biological fluids, especially plasma, consisting mainly of phospholipids, lipids, nucleic acids, peptides and proteins derived from resident cells and/or passive/active diffusion through the alveolar–capillary barrier [1,2]. A key point to understanding its diagnostic potential is that BALF in some measure collects so-called “epithelial lining fluid (ELF),” a set of soluble components responsible for the structural integrity of airspaces, gas-exchange maintenance and immune protection in the airways and alveoli. Interestingly, the protein composition of ELF, and also BALF, is affected by external factors and/or pathological conditions affecting the lung; therefore, it closely reflects the pathological status given by certain pulmonary disorders. BALF examination is, therefore, an optimal tool of ELF assessment and of diagnosis and monitoring of pulmonary diseases [3]. To this purpose, BAL testing is particularly useful for the diagnosis of “interstitial lung diseases (ILDs),” especially those of unknown etiology such as “Idiopathic Pulmonary Fibrosis (IPF)” [4]. The latter can, indeed, be defined as a chronic progressive fibroproliferative disease characterized by fibroblast and myofibroblast deposition in the alveolar walls and uninterrupted production of extracellular matrix, leading to impaired parenchyma structure and gas exchange [5,6]. As a differential diagnosis of this disorder is particularly difficult, BAL cytological analysis must be performed in combination with other diagnostic procedures. Consequently, many advances and improvements have been made to deeply improve ILDs discrimination [7]. In particular, BALF proteomics has surged ahead, now providing not only qualitative descriptive results but also clinically applicable and quantitative ones [8]. Indeed, studies of the BALF proteome have been providing new insights into pathophysiological biochemical mechanisms and suggesting novel potential biomarkers of lung diseases [3,5,9,10]. In order to fulfil this objective, many research groups have been investigating the pathophysiological role of extracellular vesicles (EVs) in lung diseases. As generally known, EVs are lipid-bound vesicles secreted by cells into the extracellular space, and they are classified into three subtypes according to size: exosomes (20–200 nm), microvesicles (200–1000 nm) and apoptotic bodies (1000–5000 nm). These structures transfer regulatory signals, mainly proteins, lipids, DNA, mRNAs and miRNAs, to target cells exploiting a remarkable cell-to-cell communication [11]. Interestingly, the composition and content of these vesicles change in the course of lung diseases, emphasizing their value as a new source of biomarkers and potential therapeutic vehicles [12]. Despite great interest in EVs’ potential, few studies have focused on their role in IPF. In detail, Njock et al. investigated exosomes from the sputum of IPF patients, demonstrating a correlation of three specific exosomal miRNAs (miR-142-3p, miR-33a-5p, let-7d-5p) with disease severity [13]. Other reports have focused on serum EVs evaluation in IPF patients; for instance, Yamada et al. proved a correlation of serum EV miR-21-5p with progression and prognosis of the disease [14]. Nonetheless, very few studies have investigated EVs in BALF from IPF subjects and, most highlighted miRNAs in EVs: for instance, Lee et al. found miRNA-rich EVs in BALF from a healthy mouse model and set up a specific isolation protocol [15], while Liu et al. investigated the expression pattern of miRNAs in exosomes from BALF of elderly IPF patients, identifying downregulation of miR-30a-5p in IPF subjects [16]. Furthermore, Martin-Medina et al. demonstrated that EVs from BALF of IPF patients act as carriers of signaling mediator WNT5A, contributing to disease pathogenesis [17]. Given these points, this study aimed at the proteome characterization of EVs from BALF of IPF patients by first setting up the EVs isolation protocol by ultracentrifugation, followed by quality control assessment by Transmission Electron Microscopy (TEM) and two-dimensional electrophoresis (2DE), in order to highlight the distinctive vesicular proteomic profile. As a further step, proteome identification was carried out by shotgun LC-MS/MS, then followed by enrichment analysis of EVs’ exclusive proteins.

## 2. Results

### 2.1. Quality Control Assessment of EVs from BALF of IPF Patients

**Figure 1:**
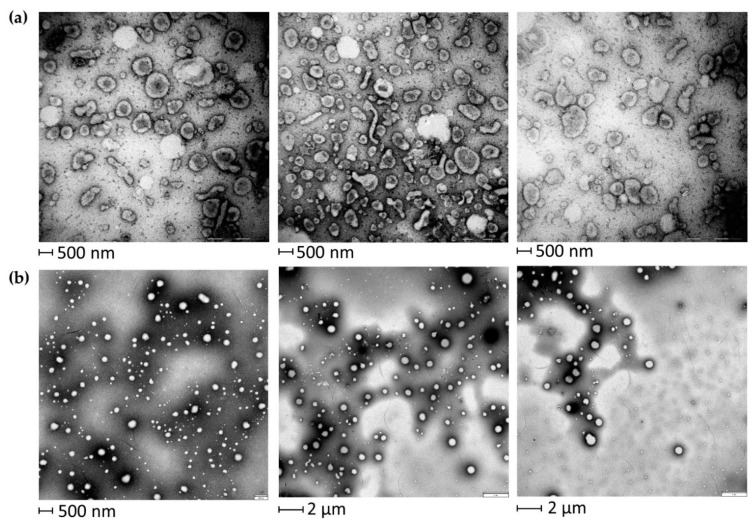
TEM of EVs from BALF of IPF patients (**a**) and of other ILD patients (**b**). EVs of both IPF and other ILD BALF samples show a variety of EVs ranging from smaller sizes (40–150 nm) to medium and larger sizes (150–2000 nm).

**Figure 2:**
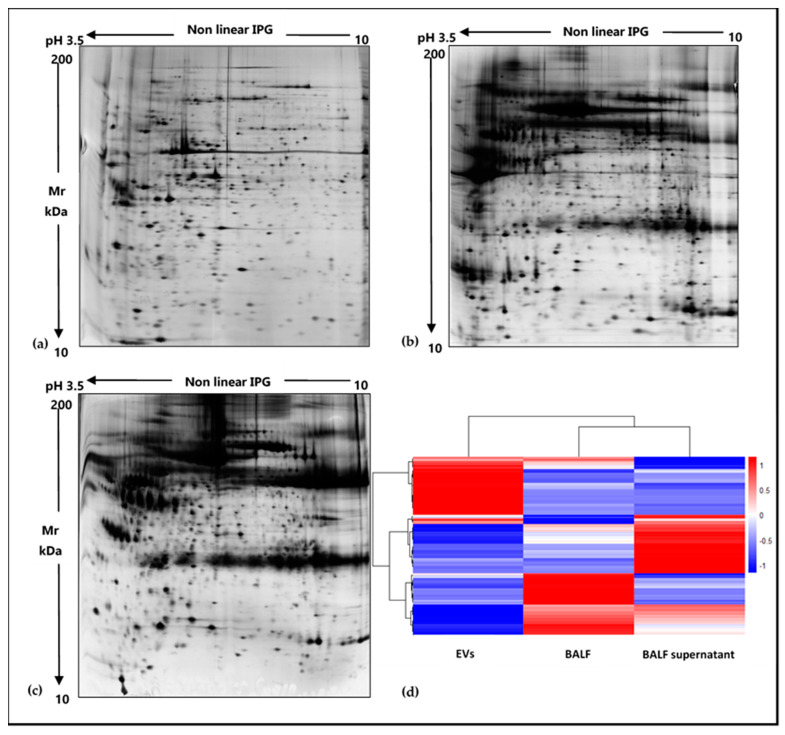
Two-dimensional gel electropherograms of BALF EVs, BALF supernatant and whole BALF and corresponding heatmap based on 2DE data sets. (**a**) 2DE gel of BALF EVs, (**b**) 2DE gel of BALF supernatant, (**c**) 2DE gel of whole BALF, (**d**) heatmap of EVs, whole BALF and supernatant, displaying variations of an abundance of 2DE spots data ranging from low abundant (blue) to high abundant (red) state.

EVs were isolated from BALF of IPF patients (Table 1) by sequential ultracentrifugation, and their purification was assessed by TEM (Figure 1). As illustrated in the TEM images of EVs from BALF of IPF patients (Figure 1a), this easily reproducible isolation technique enabled the separation of a wide size range (40–2000 nm) of vesicles with spherical morphology, sometimes assembled into small aggregates. The isolation method and the subsequent variety of isolated EVs were further assessed by performing TEM images of EVs isolated from BALF of other ILD patients. In order to further confirm the correct isolation of EVs, 2DE of EVs and of the BALF supernatant was performed. The resulting 2D gels showed patterns of proteins ranging from MW 200–10 kDa and from pH in isoelectric points 3.5–10. The EVs proteomic profile obtained (Figure 2a) was considerably different from that of the supernatant and deviated sharply from that of the whole BALF (Figure 2b,c, respectively). Conversely, 2DE gels showed a strong similarity between the whole BALF and the supernatant. To easily correlate and visualize 2DE data variations between the three components, a heatmap was performed (Figure 2d). The whole BALF and the supernatant 2DE data sets cluster together, while EVs’ data set distantly correlate with the supernatant and whole BALF. In addition, Figure 2d clearly displays that each group shows a different enriched subgroup of spots.

### 2.2. Shotgun Proteomics of BALF EVs and BALF Fluidic Portion

**Figure 3:**
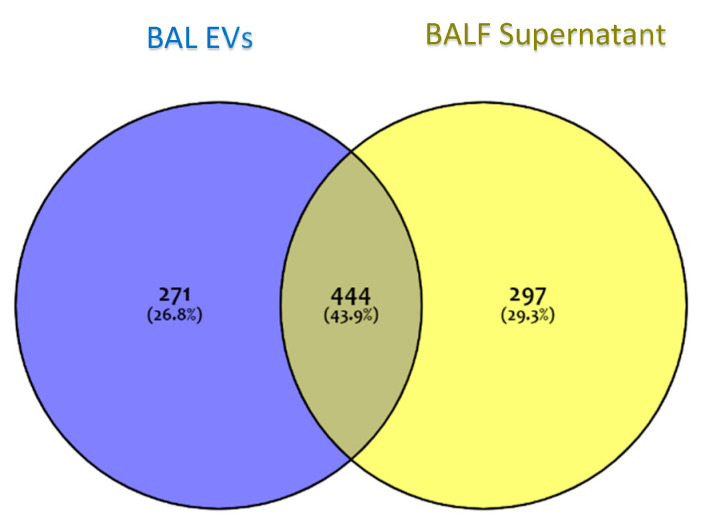
A Venn diagram of protein groups identified in EVs and in BALF supernatant. The blue area represents protein groups exclusive to EVs; the yellow area represents protein groups exclusive to BALF supernatant; grey represents protein groups common to both fractions.

The proteome of the vesicular component of BALF was obtained by the shotgun approach. The mass spectrometry proteomics data have been deposited to the ProteomeXchange Consortium via the PRIDE [18] partner repository with the dataset identifier PXD025590. The proteome of the vesicular component of BALF was obtained by the shotgun approach. In order to identify those proteins specific to the vesicular fraction, a Venn diagram was performed submitting the proteome lists of EVs and of the complementary supernatant, which accounts for 715 and 741 proteins, respectively (Figure 3). As the Venn diagram shows, 271 proteins (26.8%) were exclusive to EVs, 297 (29.3%) were exclusive to the BALF supernatant, and 444 (43.9%) were common to both.

**Figure 4:**
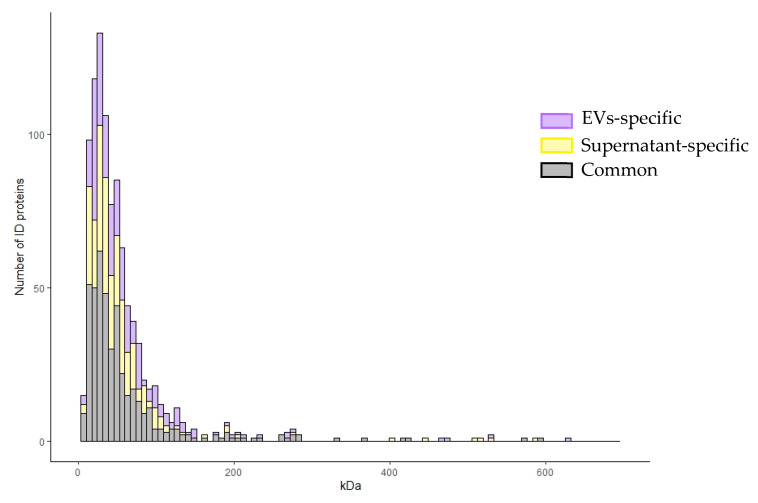
Frequency distribution of EVs identified proteins. Distribution of identified proteins, exclusive or common to the vesicular and supernatant fractions, according to their molecular weight on the *x*-axis and to their frequency on the *y*-axis.

Furthermore, Figure 4 shows the distribution of identified proteins, exclusive or common to the vesicular fraction and the supernatant, according to their molecular weight on the *x*-axis and to their frequency on the *y*-axis. The graph shows that exclusive vesicular proteins prevalently and more frequently distribute in the molecular weight range of 0–180 kDa, with respect to the supernatant exclusive and common ones. In particular, exclusive vesicular proteins distribute at a larger extent at a low molecular weight range. For these reasons, we focused on the exclusive vesicular proteomic dataset.

**Figure 5:**
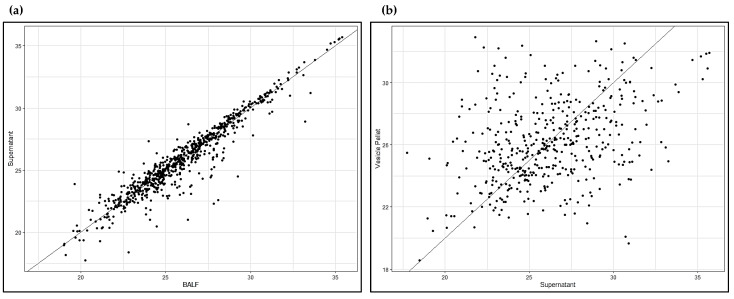
Scatter plots. (**a**) Whole BALF vs. BALF supernatant; (**b**) BALF supernatant vs. EVs.

Supplementary Table S1 reports the EVs protein list. Specific vesicular markers (in bold in Table S1), such as TSG101, CD9, CD63, CD151, TSPAN1, LAMP1, Rab family proteins, ARF6, ADAM10, SDCBP, HSP90AA1, HSP90B1, ANXA11, HSP90AB1 and FLOT1, were identified, further confirming good quality isolation of EVs from our BALF samples. To highlight the distinctive proteomic profile of the EVs data set, we conducted a scatter plot analysis of mass spectrometry data, this time including the whole BALF data set as well. Figure 5a shows the scatter plot with the whole BALF data set on the *x*-axis and the BALF supernatant data set on the *y*-axis, indicating a strong positive linear correlation. Figure 5b shows a scatter plot with the BALF supernatant data set on the *x*-axis and the EVs data set on the *y*-axis, indicating a weak positive linear correlation.

### 2.3. MetaCore Enrichment Analysis

**Figure 6:**
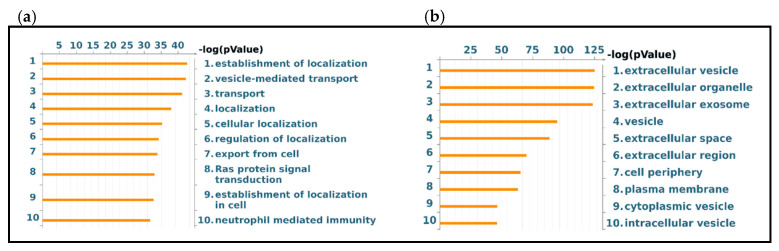
GO Enrichment analysis of BALF EVs. GO Biological Processes (**a**) and GO Localization (**b**) of protein groups exclusive to EVs.

**Figure 7:**
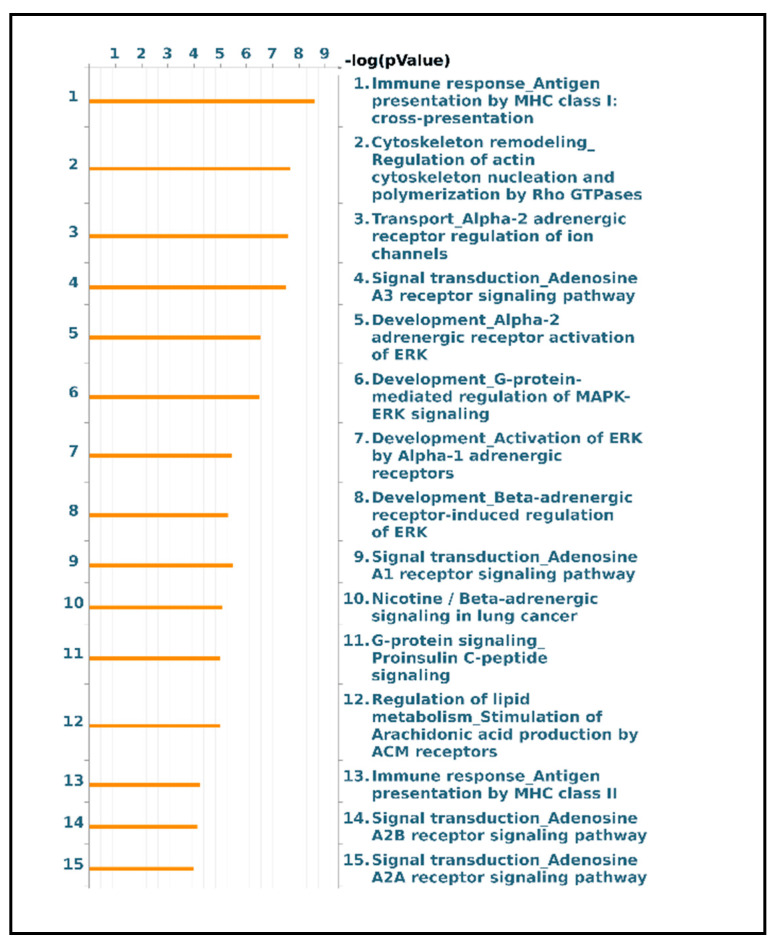
Major pathway maps of EVs exclusive proteins for the major 15 molecular pathways enriched in EVs dataset.

Functional analysis of the protein groups identified in EVs was performed using the MetaCore software suite. Gene Ontology enrichment analysis of biological processes and localization was carried out to obtain a wider overview of the experimental data sets. Figure 6a shows the 10 most significant biological processes enriched from the exclusive EV mass spectrometry data, including the establishment of localization, vesicle-mediated transport, localization, export from cell and Ras protein signal transduction. Figure 6b reports the ten most significant GO localization terms enriched from the proteins exclusive to vesicular sample, including extracellular vesicle, exosome, extracellular space, plasma membrane, cell periphery and intracellular vesicle. In a second step, the functional analysis was implemented, performing a pathway analysis of the EVs data set. The 15 most relevant pathway maps are listed in Figure 7 (complete list in Supplementary Figure S1). As Figure 7 shows, the EVs exclusive data set is associated with major involvement in pathways related to immune response through antigen presentation, cytoskeleton remodeling, alpha-2 adrenergic receptor regulation of ion channels, adenosine receptors signaling pathways, beta-adrenergic signaling in lung cancer, proinsulin C-peptide signaling, G protein-coupled receptor signaling in lung cancer and lipid metabolism (complete list in Supplementary Table S2). As Figure 6 shows, the EVs exclusive data set is associated with major involvement in most of the relevant pathways, as it is related to immune response through antigen presentation, cytoskeleton remodeling, alpha-2 adrenergic receptor regulation of ion channels, adenosine receptors signaling pathways, beta-adrenergic signaling in lung cancer, proinsulin C-peptide signaling and G protein-coupled receptor signaling in lung cancer (complete list in Supplementary Table S2). 

## 3. Discussion

In the long-standing task of finding specific, easily detectable and reliable biomarkers of pulmonary diseases, much work has been dedicated to alternative biofluids such as BALF [18]. Many studies on the BALF proteome have being made targeting widespread diseases such as lung cancer [19,20], cystic fibrosis [21,22], asthma [23,24] and chronic obstructive pulmonary disease [25,26]. Nonetheless, BALF proteomics applied to the study of ILDs could provide reliable new results. In particular, growing efforts to investigate the role of EVs in the physiopathology of ILDs and their possible therapeutic applications have become more common in the scientific community [27,28], especially applied to IPF [13,14,17]. The few recent studies on EVs in BALF from IPF patients have mainly focused on nucleic acid content, such as miRNAs [16]. For this reason, proteomic studies could provide alternative insights into the disease [17]. The main aim of our analysis was to characterize the proteome of the vesicular component of BALF, highlighting that pre-fractioning of BALF samples allows identifying low abundant proteins, not easily detectable by whole BALF analysis. For this purpose, we chose ultracentrifugation as the technique to isolate EVs [29], and their purification was assessed by TEM [30], 2DE and LC-MS/MS. First, electron microscopy showed a broad range of EVs, ranging from small (40–150 nm in size) to larger particles (150–2000 nm in size), suggesting that this isolation method was effective in separating various classes of vesicles. Secondly, 2DE of the vesicular fraction and of the supernatant of BALF was performed and demonstrated considerable differences in the two fractions. The protein pattern of the supernatant was similar and comparable to that of BALF, while no evidence of protein species typical of BALF could be observed in the EV profile. We then identified proteins by a shotgun approach using LC-MS/MS. Scatter plot analysis of mass spectrometry data of the two fractions confirmed our 2DE-proteomic profiles. Interestingly, these results confirmed how distinct the EV proteome was from those of the whole BALF and supernatant. In addition, the prevalent and more frequent distribution of exclusive vesicular proteins in the 0–180 kDa weight range provide evidence that this methodological approach is effective in isolating not only low abundant but also low molecular weight proteins, which result to be even much more difficult to be detected by classic experimental approaches. Our findings, therefore, support the importance of pre-fractioning procedures in proteomic technologies as they enable us to overcome the limits of identifying low abundant species in complex samples, such as BALF. Most of the EVs proteins are, indeed, not easily detectable in BALF samples both by 2DE/MS and LC-MS/MS proteomic approaches, as they are usually hidden by major protein species; therefore, our study confirms the importance of isolating and studying separately the vesicular and the supernatant fractions as a source of potential biomarkers. 

We implemented this characteristic proteomic profile of the BALF vesicular component by evaluating the protein content and functions in specific biological processes, localization and molecular pathways. Enrichment of GO biological processes provide evidence of EVs predominant involvement in mechanisms of transport, localization and signal mediation, confirming their key roles in intercellular communication under physiological and pathological conditions [31], while the enrichment of GO localization terms fully confirms that EVs identified proteins mainly collocate in the extracellular spaces, extracellular and intracellular vesicles and plasma membrane.

Pathway analysis of exclusive EVs proteins suggested interesting results, as, to the best of our knowledge, they did not come to light from previous published proteomic studies of BALF of IPF subjects. In support of this, Khan et al. performed a useful meta-analysis of all proteomic studies on IPF, reporting also a state-of-the-art classification of BALF proteomics studies. This analysis highlights an interesting trend as all these studies, although using different proteomic approaches and mediated by distinct protein factors, show the recurrence of the same dysregulated molecular pathways [32]. One major molecular pathway is oxidative stress response: Magi et al. reported an up-regulation of antioxidant proteins in IPF, such as antioxidant peroxisomal enzyme and thioredoxin peroxidase 2 [33], later supported by Rottoli et al., who reported an upregulation of other antioxidants, such as ceruloplasmin, and an increased concentration of protein carbonyls in IPF BALF, suggesting an extended oxidative proteomic pattern [34,35]. Furthermore, the involvement of oxidative stress and altered antioxidant defense system has been often reported in ELF of IPF patients, as reported by Cameli et al. [36]. Furthermore, both also highlighted in IPF BALF an up-regulation of inflammatory mediators, such as macrophage migration inhibitory factor (MIF) and calgranulin B (S100A9) [33,34,35], which were further evaluated and confirmed to be more abundant in BALF of IPF patients by Bargagli et al. [37,38] and Hara et al. [39]. Landi et al. provided major contributions to IPF pathogenetic mechanisms by functional proteomic analysis reporting the involvement of several molecular pathways. Some of those are protein folding, Slit-Robo signaling, hypoxia response, blood coagulation system and complement-mediated immune response and angiotensin system [5,9]. Furthermore, Carleo et al. confirmed and implemented previous results suggesting the involvement of the Wnt-β-catenin transduction signaling, lung carcinogenesis pathway and a protease/antiprotease imbalance in IPF BALF patients with acute exacerbations, in addition to the involvement of ER stress, ion homeostasis and wound healing processes [6,40]. The most suggested molecular pathway is represented by pro-fibrotic mechanisms mediated by several factors, such as osteopontin, matrix-metalloproteinases, CCL24, CXCL7 and CCL18, as reported by Foster et al. by shotgun proteomic analysis of IPF BALF [41].

Interestingly, our analysis highlighted various molecular pathways, probably mediated by low abundant proteins, which are hardly uncovered as hidden by the abundance of major protein species.

Remarkably, pathways analysis of EVs proteins showed that they are prevalently involved in antigen presentation by MHC class I and II, cytoskeleton remodeling, adenosine signaling, adrenergic signaling, G protein signaling, specific G protein C-peptide signaling and lipid metabolism. C-peptide (proinsulin), prevalently studied in diabetes, exerts its biological activities via a specific G-protein coupled receptor also expressed on endothelial cells and fibroblasts [42]. Its signaling involves ERK1/2, PI3K-Akt, PKC, eNOS and NF-kB, well-known factors in TGF-β signaling [43,44], the key regulator of fibrosis, thereby suggesting C-peptide involvement in fibrogenic processes [45]. Indeed, recent studies show an association of C-peptide with fibrosis progression in different pathologies [46,47]. Some studies have reported the association of C-peptide with the transcription factor peroxisome proliferator-activated receptor-γ (PPARγ) [48], whose modulation balances adipogenesis and fibrogenesis [49], in line with the concept of metabolic dysregulation as an additional impacting cause of fibrosis, especially in IPF [45,49].

Given also the identification in EVs of monoacylglycerol lipase (MAGL), a pro-inflammatory enzyme catalyzing the arachidonic acid production, pathway analysis suggests the involvement of lipid metabolism mediated by the stimulation of the production of arachidonic acid. Costola-de-Souza et al. report interesting results according to which MAGL inhibition displays anti-inflammatory and protective effects during acute lung injury in mice [50], while a more recent study of Habib et al. demonstrated that MAGL inhibitors have a powerful impact on liver fibrosis as it delays fibrosis progression and promotes its regression [51].

Our identification of several Rho GTPases in EVs suggests their potential action on cytoskeleton remodeling by mediating actin filament rearrangement via ROCK [52]. Indeed, interesting studies report that ROCK signaling pathways are involved in myofibroblast differentiation and fibrogenic processes, especially pulmonary fibrosis such as IPF [40,53,54]. Curiously, given the growing attention to Wnt signaling in the regulation of cellular adhesions and its involvement in IPF pathogenic mechanisms, Franco et al. reported that Rho GTPases’ regulation modulates cell migration and polarity via a β-catenin-independent Wnt pathway [55]. Another remarkable cytoskeleton-related protein detected in EVs is profilin, which triggers fibrogenic pathways such as PI3K-Akt and ERK 1/2 [56]. 

Our results indicate the involvement of another interesting pathway related to fibrogenesis, which has recently attracted attention: the adenosine signaling pathway. Adenosine exerts its functions by binding to G-protein coupled receptors A2A and A2B, leading to fibroblast activation and collagen synthesis [57]. Indeed, several studies already report a correlation between A2B adenosine receptor (A2BAR) activation and regulation of inflammation and fibrosis in IPF, specifically indicating macrophages as major mediators [58,59]. Some signal transduction factors of this signaling, such as PKA, are detected in EVs. Curiously, a recent study demonstrated the key role of A2BAR in the modulation of the EMT process in IPF by two signaling pathways, cAMP/PKA and MAP/ERK [60]. Our enrichment analysis suggests a direct link between PKA and CREB1 activation, inducing VEGF-A transcription, a major player in IPF onset [61].

We also found another molecular pathway whose relation to IPF pathogenesis is not often considered: the alpha- and beta-adrenergic systems. Rassler B. demonstrated that continuous stimulation of beta- and especially alpha-adrenergic signaling in rats leads to pulmonary fibrosis. Adrenergic-stimulated histological lung fibrosis is associated with a remarkable increase in TGFβ1, collagen I, MMP-2 and TIMP-2 mRNA expression, suggesting a link between adrenergic stimulation, the up-regulation of ECM molecules and the promotion of fibrotic processes [62].

Although these altered molecular pathways were detected by proteins directly identified in BAL samples, our results provide evidence of a potential wider systemic involvement. In other words, altered vesicular protein-mediated processes may be regulated in situ by cellular protein release and by systemic circulating molecules, which may be released in the lung environment by plasma exudation into epithelial lining fluid. Likewise, vesicular proteins may be of systemic origin as well as pulmonary origin. Altered systemic metabolic pathways could therefore use EVs as a communication system to induce a specific pro-fibrotic response in the lung environment, leading to IPF.

## 4. Materials and Methods

### 4.1. Population

Male IPF patients, mean age 69 ± 5 years, 2 ex-smokers and 1 never smoker, were enrolled in the study. The patients were diagnosed according to ATS/ERS guidelines at the Medizinische Hoschschule Pneumology Clinic (Hannover, Germany). Demographic data and smoking habits of the three patients used for this sample characterization were reported in Table 1 together with other clinical data such as FVC, DLCO and GAP percentages according to ATS/ERS guidelines. The diagnosis of IPF was formulated in the context of the multidisciplinary discussion. After informed consent of the patients, BAL was performed for diagnostic purposes in order to exclude other ILDs. Samples were provided by the research group directed by Prof. Antje Prasse at Fraunhofer ITEM (Hannover, Germany).

### 4.2. EVs Isolation from BALF 

Human IPF BALF samples were centrifuged at 800× *g* for 5 min at 4 °C, as a routine procedure to separate BALF and cell components [63,64]. In particular, BALF samples from IPF patients were prepared for the analysis, specifically using 15 mL per each as starting volume. First, BALF samples were centrifuged at 12,000× *g* for 45 min at 4 °C. The pellet was discarded, and the supernatant was collected in ultracentrifuge tubes. Then, supernatants were centrifuged at 110,000× *g* for 2 h at 4 °C (Beckman Coulter Optima XE, Type 70 Ti Fixed-Angle Titanium Rotor, Beckman Coulter Life Sciences, Brea, CA, USA). At this step, the supernatant was collected in a new tube and stored on ice, as this fraction was the complementary portion of BALF whole fluid without EVs (BALF supernatant). Conversely, the pellet was resuspended in PBS and filtered into a new ultracentrifuge tube through a 0.22 µm filter and centrifuged at 110,000× *g* for 70 min at 4 °C. Following this, the supernatant was discarded, and the pellet was resuspended in PBS and centrifuged again at 110,000× *g* for 70 min at 4 °C; at this point, the pellet containing BALF EVs was transferred into a new Eppendorf [29]. The concentration of BALF EVs was detected by NanoDrop (NanoDrop ND-1000 spectrophotometer).

### 4.3. TEM

As the first checkpoint of isolation of BALF EVs, TEM was performed by Dr. Eugenio Paccagnini and Dr. Mariangela Gentile, members of the research group directed by Prof. Pietro Lupetti of the Life Sciences Department at the University of Siena. In detail, about 3 μL of EV fraction was loaded on a 300 mesh formvar coated copper grid for 2 min. After blotting the excess, the grid was negatively stained with 2% aqueous ammonium molybdate for 30 s and analyzed using a Thermo Fisher Scientific Tecnai G2 Spirit transmission electron microscope operating at 120 kV equipped with an EMSIS Veleta 2048 × 2048 CCD camera.

### 4.4. Samples Preparation for 2DE Analysis

Dialysis of the BALF EV and supernatant was performed against four changes of distilled water at 4 °C for 12 h to eliminate salts. Samples were lyophilized and dissolved in lysis buffer (8 M urea, 4% *w*/*v* 3-[(3-cholamidopropyl) dimethylammonia]-1-propanesulfonate hydrate (CHAPS), 40 mM Tris base, 1% *w*/*v* dithioerythritol (DTE) and trace amounts of bromophenol blue).

Before adding bromophenol blue, the protein concentration of the BALF supernatant was determined by Bradford assay [65] in order to load 60 µg of protein per gel, while EVs’ total protein content was used.

### 4.5. 2D-Electrophoresis

2DE was carried out using the Immobiline polyacrylamide system on a preformed immobilized nonlinear pH gradient from pH 3 to 10, 18 cm in length (Cytiva, formerly GE Healthcare, Uppsala, Sweden). The 2D run was performed using Ettan™ IPGphor™ system (Cytiva, formerly GE Healthcare, Uppsala, Sweden) at 16 °C, applying the following electrical conditions: 200 V for 8 h, from 200 to 3500 V in 2 h, 3500 V for 2 h, from 3500 to 5000 V in 2 h, 5000 V for 3 h, from 5000 to 8000 V in 1 h, 8000 V for 3 h, from 8000 to 10,000 V in 1 h, 10,000 V, for a total of 90,000 VhT (total Volts per hour). Gel strips were rehydrated with lysis buffer and traces of bromophenol blue overnight at room temperature; then, 0.2% carrier ampholyte was added to samples, and the run was performed by cup loading, with the cup placed at the cathodic end of the strips. After the first dimensional run, strips were equilibrated in 6 M urea, 2% *w*/*v* SDS, 2% *w*/*v* DTE, 30% *v*/*v* glycerol and 0.5 M Tris–HCl pH 6.8 for 12 min and for a further 5 min in 6 M urea, 2% *w*/*v* SDS, 2.5% *w*/*v* iodoacetamide, 30% *v*/*v* glycerol, 0.5 M Tris–HCl pH 6.8 and a trace of bromophenol blue. Then, the second dimension was performed on 9–16% SDS polyacrylamide linear gradient gels (18 × 20 cm × 1.5 mm) at 40 mA/gel constant current and 9 °C until the dye reached the bottom of the gel. Gels were finally stained with ammoniacal silver nitrate. Gels were then digitalized using the Image Scanner III laser densitometer supplied with the LabScan 6.0 software (GE Healthcare), image analysis was performed using Melanie Classic 9.0 software and normalization of the 2DE data was performed. The heatmap was performed on normalized spot volumes (%V) using RStudio Desktop 1.1.463 (Integrated Development for RStudio, Inc., Boston, MA, USA, https://www.rstudio.com (accessed on 1 April 2021).

### 4.6. MS-Preparative SDS-PAGE

For shotgun proteomic analysis, BALF whole fluid of the patients was also prepared, and BALF whole fluid and BALF supernatant underwent cold acetone precipitation (1:4) overnight at −20 °C. They were then centrifuged at 4542× *g* for 10 min at 4 °C. The pellet was resuspended in acetone and centrifuged again at 15,000× *g* for 10 min at 4 °C. At this step, the three components of each sample (whole BALF, BALF supernatant and EVs) were solubilized in a denaturating solution composed of 8 M urea and 4% *w*/*v* CHAPS, and their protein concentration was determined by Bradford assay [65]. 

MS-preparative SDS-PAGE was carried out using pre-cast 12% polyacrylamide gels (Criterion™ XT Bis-Tris Protein Gel, Bio-Rad, Hercules, CA, USA) in a Criterion™ Vertical Electrophoresis Cell (Bio-Rad, Hercules, CA, USA) with the following voltage conditions: 60 V for stacking gel and 120 V for separating gel. The amount of protein loaded was 50 µg for BALF and BALF supernatant and the total protein content for EVs. Samples were centrifuged, and the XT sample buffer and XT reducing agent (Bio-Rad, Hercules, CA, USA) were added to samples, which were then held at 95 °C for 5 min. After that, the proteins were alkylated by adding 40% acrylamide at a final concentration of 2%, and the samples were loaded in the gel. After the run, the gel was incubated in 50% *v*/*v* methanol and 10% *v*/*v* glacial acetic acid fixing solution for 1 h under gentle agitation. It was then stained in Coomassie Blue solution composed of 0.1% *w*/*v* Coomassie Brilliant Blue R-250, 50% *v*/*v* methanol and 10% *v*/*v* glacial acetic acid for 20 min under gentle agitation.

### 4.7. Preparation of Samples for LC-MS/MS

Protein bands were cut out and minced into 3 1 mm pieces. The pieces were destained twice in 50% *v*/*v* acetonitrile (ACN)/20 mM Ammonium Bicarbonate (ABC) at 37 °C under shaking (Thermomixer, Eppendorf AG, Hamburg, Germany) at 700 rpm for 30 min. The gel pieces were then dehydrated in 100% ACN at room temperature for 10 min, and the solvent was removed in a vacuum centrifuge (Speedvac, Thermo Fischer ScientificTM, Waltham, MA, USA) for 30 min. Then, a solution of 10 ng/µL trypsin in 10% *v*/*v* ACN/20 mM ABC solution for protein digestion was added to the gel pieces, which were rehydrated on ice for 60 min; then, covered with 10% *v*/*v* ACN/20 mM ABC solution and digested overnight at 37 °C under shaking at 350 rpm. Digestion was then stopped by adding 50% *v*/*v* ACN/5% *v*/*v* trifluoroacetic acid (TFA) solution. The gel pieces were incubated at 24 °C under shaking at 700 rpm for 30 min. The supernatant containing peptide extracts was collected into a new vial and dried in a vacuum centrifuge for 30 min. A 50% *v*/*v* ACN/0.5% *v*/*v* TFA solution was added, and the mixture was incubated under the same previous conditions. The supernatant was pooled with the previous one and dried again in the vacuum centrifuge for 30 min. Then, 100% ACN was added to gel pieces, and they were incubated at 24 °C under shaking at 700 rpm for 20 min. Finally, the supernatant was collected and dried in a vacuum centrifuge for 3 h.

### 4.8. LC-MS/MS Analysis and Protein Identification

Peptides of mono-dimensional gel digestion were analyzed by LC-MS/MS. Dried samples were dissolved in 2% *v*/*v* ACN/0.1% *v*/*v* TFA solution and incubated at 24 °C under shaking at 350 rpm for 30 min, then centrifuged at 20,000× *g* for 30 min at room temperature and the supernatants were transferred to an LC sample vial. An appropriate amount of each sample was injected into a Dionex Ultimate 3000 high-performance LC system (Thermo Fisher Scientific, Waltham, MA, USA). Peptides were loaded on a C18 trap column (2 cm long, 75 µm i.d., Acclaim PepMap, Dionex) at 6 µL/min and washed with 0.1% *v*/*v* TFA loading buffer. After 5 min, the trap column was switched in line with the C18 nanoflow separation column (50 cm long, 75 µm i.d., Acclaim PepMap, Dionex) and the peptides were eluted with a linear gradient of elution buffers A (0.1% *v*/*v* formic acid) and B (80% *v*/*v* acetonitrile, 0.1% *v*/*v* formic acid) at 250 nl/min. The LC system was connected to the nanoESI source of an LTQ Orbitrap Lumos Mass Spectrometer (Thermo Fisher Scientific, USA). The Orbitrap mass analyzer recorded the survey scans selecting the most intense precursor ions of charge state ≥ 2 for collision-induced fragmentation with a normalized collision energy of 38%. Fragments were scanned out in the Orbitrap mass analyzer in centroid mode, and the raw data were processed with MaxQuant software (Version 1.6.50, https://maxquant.net/maxquant/ (accessed on 20 June 2020). For peptide identification, MS/MS spectra were searched against human entries in the UniProtKB/Swiss-Prot database and were considered to be identified for false discovery rates (FDR) on protein and peptide level ≤ 0.01. Oxidation of methionine residues, N-terminal acetylation, deamidation of asparagine and glutamine residues and propionamidation of cysteine residues were selected as variable modifications. A maximum of two missed cleavages was accepted. A minimum ratio count of one unique or razor peptide was required for quantification. The protein groups identified were processed for statistical purposes using Perseus software (Version 1.5.2.6, https://maxquant.net/perseus/ (accessed on 20 June 2020). Shotgun experiments were performed in collaboration with Dr. Alfonso Carleo, a member of the Prof. Antje Prasse research group at Fraunhofer ITEM (Hannover, Germany).

### 4.9. Enrichment Analysis

Enrichment analysis was performed by submitting the gene names of identified proteins to the MetaCore 6.8 network building tool (http://portal.genego.com (accessed on 1 April 2021), Clarivate Analytics, Philadelphia, PA, USA). Specifically, we first performed enrichment analysis by GeneGo ontology biological processes, localization and then pathway maps analysis. The software establishes a hierarchical list of pathway maps, prioritized according to their statistical significance (*p* ≤ 0.001), and each is equivalent to a canonical map that has multiple sequential steps of interactions, defining a well-established signaling mechanism. Each step is also well-defined, experimentally validated and accepted in the research field.

## 5. Conclusions

In conclusion, EVs’ isolation and separation from its complementary fraction turned out to be extremely useful as they shed light on unexpected and infrequently IPF-associated molecular pathways, which might be hardly identifiable by classic experimental approaches. Interestingly, the protein content of BALF EVs regulates distinct molecular pathways, suggesting a particular new disease scenario in which systemic and metabolic dysregulation may be a cause and/or a consequence of IPF development. Vesicular proteins may indeed potentially cooperate with proteins in the complementary fractions to the pathogenesis and maintenance of the disease through pro-fibrotic and pro-inflammatory signals. These findings support the much-needed improvement in proteomic analysis in fractioning complex samples, allowing low abundant proteins to emerge and to unveil particular signaling pathways. This might represent a valuable starting point for further study in a larger cohort of patients where IPF samples could be compared with other ILDs and control samples in order to provide new insights into the pathophysiology of the disease and biomarkers discovery.

## Figures and Tables

**Table 1:** Demographic and clinical data of enrolled patients. Table reports the diagnosis, the gender, age, smoking habit and clinical information (FVC, DLCO and GAP) of enrolled patients.

Diagnosis	Gender	Age	Smoking	FVC	DLCO	GAP
IPF	Male	74	ex	62	43	2
IPF	Male	63	ex	39	22	3
IPF	Male	69	never	50	32	3

## Data Availability

Requests for further information about resources, reagents and data availability should be directed to the corresponding author.

## Supplementary Materials

The following are available online at https://www.mdpi.com/article/10.3390/ijms22115696/s1, Figure S1: Pathway maps enrichment of EVs proteins from IPF BALF. Complete list of the 50 most statistically significant pathway maps of vesicular proteins from IPF BALF; Table S1: MS complete protein lists of vesicular proteins, reported by gene name and protein name, identified by LC-MS/MS. Vesicular markers are highlighted in bold; Table S2: Pathway maps of proteins exclusive to EVs. Excel report of the 50 most significantly enriched pathway maps of proteins exclusive to EVs: maps, *p*-value, FDR, number of proteins associated, network objects.
