# Supplementary material for: Proteome Characterization of BALF Extracellular Vesicles in Idiopathic Pulmonary Fibrosis: Unveiling Undercover Molecular Pathways
Source: Int J Mol Sci. 2021 May 27;22(11):5696. doi: 10.3390/ijms22115696 (PMC8199247; doi:10.3390/ijms22115696)
Supplement: Supplementary file 1 [file ijms-22-05696-s001.zip › Supplementary Figure 1.pdf]

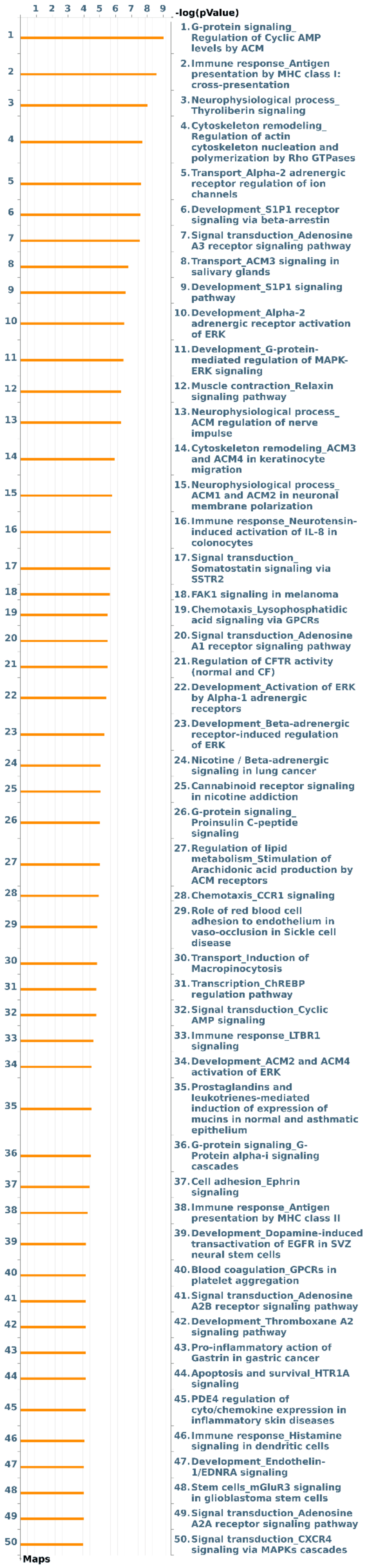

Figure S1. Pathway maps enrichment of EVs proteins from IPF BALF

Complete list of 50 most statistically significant pathway maps of vesicular proteins from IPF BALF
